# Effects of Web-Based Orofacial Myofunctional Therapy on Hyoid Bone Position in Adults with Mild to Moderate Obstructive Sleep Apnea: Evidence from an Estonian Substudy of a Randomized Controlled Trial

**DOI:** 10.3390/jcm15010257

**Published:** 2025-12-29

**Authors:** Andres Köster, Anh Dao Hoang, Andrey Dashuk, Heisl Vaher, Katrin Sikk, Triin Jagomägi

**Affiliations:** 1 Institute of Dentistry, University of Tartu, 50405 Tartu, Estonia; anh.dao.hoang@ut.ee (A.D.H.); andrey.dashuk@kliinikum.ee (A.D.); triin.jagomagi@ut.ee (T.J.); 2 Department of Rehabilitation Medicine, North Estonia Medical Centre, 13419 Tallinn, Estonia; 3 Faculty of Odonto-Stomatology, Hue University of Medicine and Pharmacy, Hue University, Hue 10000, Vietnam; 4 Department of Otorhinolaryngology, Fertilitas Private Hospital, 10614 Tallinn, Estonia; h.vaher@fertilitas.ee; 5 Institute of Clinical Medicine, University of Tartu, 50405 Tartu, Estonia; katrin.sikk@regionaalhaigla.ee; 6 Department of Neurology, North Estonia Medical Centre, 13419 Tallinn, Estonia

**Keywords:** obstructive sleep apnea, orofacial myofunctional therapy, hyoid bone, cephalometry, cone-beam computed tomography, upper airway, randomized controlled trial

## Abstract

**Background**: Orofacial myofunctional therapy (OMT) is an emerging adjunctive treatment for obstructive sleep apnea (OSA), but its effects on upper airway structural support, particularly the hyoid complex, are not well defined. This study assessed the short-term effects of OMT on hyoid bone position and sleep-related indices in adults with mild to moderate OSA. **Methods**: In this assessor-blinded randomized controlled trial (ClinicalTrials.gov Identifier: NCT06079073), 13 adults with mild to moderate OSA were randomized to a 12-week web-based OMT program (*n* = 9) or a waitlist control group (*n* = 4). Cone-beam computed tomography (CBCT) and three-night home sleep testing were performed at baseline and follow-up. The primary outcome was change in axis-based cephalometric hyoid position measures; secondary outcomes included sleep parameters such as the apnea–hypopnea index (AHI). **Results**: No significant within- or between-group differences were observed in AHI, oxygen desaturation index, or mean nocturnal SpO_2_ after 12 weeks (all *p* > 0.05). However, several cephalometric variables showed significant between-group differences. The waitlist group exhibited greater posterior–inferior hyoid displacement than the OMT group, with large effect sizes across multiple vector measures (all *p* ≤ 0.045; r = 0.56–0.66). Posterior and inferior hyoid displacement was associated with higher AHI and lower SpO_2_, whereas increased lower pharyngeal airway width was associated with lower AHI. **Conclusions**: Short-term OMT did not improve sleep-disordered breathing indices but was associated with stabilization of hyoid bone position. These findings suggest that structural stabilization may precede functional improvement and highlight the clinical relevance of vector-based hyoid analysis.

## 1. Background/Objectives

Obstructive sleep apnea (OSA) is characterized by recurrent collapse of the upper airway during sleep, typically associated with reduced oropharyngeal muscle tone and altered craniofacial morphology [1]. Continuous positive airway pressure (CPAP) remains the gold standard treatment; however, adherence is suboptimal, and up to 50% of patients discontinue therapy within the first year [2].

Orofacial myofunctional therapy (OMT) consists of targeted exercises to strengthen and coordinate the orofacial and oropharyngeal musculature. It has emerged as a promising adjunctive intervention for OSA management. Multiple clinical trials and meta-analyses have demonstrated that OMT improves upper airway patency and reduces snoring intensity. It can also substantially decrease the apnea–hypopnea index (AHI), with meta-analytic data showing reductions of approximately 50% in adults and more than 60% in children. These improvements are often accompanied by better oxygen saturation and reduced daytime sleepiness [3,4,5,6,7,8].

Despite growing evidence of OMT’s clinical efficacy, its underlying structural mechanisms remain poorly defined. This is particularly true for the role of the hyoid bone, a central stabilizer of the upper airway. Winter and colleagues recently proposed the Hyoid Slump Hypothesis, which posits that caudal (downward) displacement of the hyoid bone elongates the collapsible segment of the upper airway, thereby increasing its vulnerability to obstruction [9]. This perspective reframes the understanding of OSA. It shifts the concept from a purely soft-tissue disorder toward one involving both neuromuscular and skeletal determinants of airway collapsibility.

Within this context, OMT may function as a neuromuscular intervention that enhances muscle tone, strength, and coordination. It may also serve as a structural stabilizer that prevents downward or posterior displacement of the hyoid bone. This randomized controlled pilot study investigated the effects of a web-based OMT program on hyoid bone position in adults with mild to moderate OSA. The study also tested key predictions of the Hyoid Slump Hypothesis and explored anatomical mechanisms underlying OMT’s potential therapeutic benefits [9,10].

## 2. Materials and Methods

### 2.1. Study Design and Ethics Ethical Approval

The Orofacial Myofunctional Therapy for Obstructive Sleep Apnea (OMTaOSA) trial was an assessor-blinded, parallel-group randomized controlled study conducted within the Sleep Revolution project (European Union Horizon 2020 program, grant No. 96549). The study protocol was approved by the Regional South-Eastern Committee for Research Ethics (522434, August 2023). Approval was also granted by the Akershus University Hospital Data Protection Officer (2023_61) and by the Research Ethics Committee of the University of Tartu (381/M-12, September 2023). The trial was registered on ClinicalTrials.gov (NCT06079073) and conducted in accordance with the Declaration of Helsinki. Additional details on ethical approvals and study oversight are provided in the Back Matter section.

### 2.2. Participation and Randomization

A total of 104 Norwegian and Estonian adults with mild to moderate obstructive sleep apnea (OSA) were enrolled and randomized (1:1) into the intervention and control groups using sex-stratified block randomization to ensure balanced allocation. Both groups completed three-night home sleep studies at baseline and after the three-month intervention period. The primary outcome was the change in apnea–hypopnea index (AHI), while secondary outcomes were pre-registered on ClinicalTrials.gov. Outcome assessors and sleep scorers were blinded to treatment allocation [11].

### 2.3. Estonian Subsample and Participant Selection

Eligibility Criteria: This Estonian imaging subsample included adults (age ≥ 18 years) with suspected or confirmed mild to moderate OSA (respiratory event index < 30 events/hour on polygraphy, confirmed by polysomnography) who had no prior or current treatment with an oral appliance or positive airway pressure. Participants were required to have a body mass index < 30 kg/m^2^; ability to breathe through the nose; adequate oral opening (≥50% of maximal interincisal opening with the tongue tip positioned at the maxillary incisive papillae); all frontal teeth present from second premolar to second premolar in both dental arches; no botulinum toxin administration to facial muscles within the previous 3 months; completion of ≥70% of electronic sleep diary days during pre-screening; and willingness to undergo cone-beam computed tomography imaging at baseline and at the 12-week follow-up. Participants were excluded if they had severe OSA (apnea–hypopnea index ≥ 30 events/hour on polysomnography), medical or psychiatric conditions that could interfere with the study protocol, pregnancy, contraindications to CBCT imaging, or inability to attend both imaging appointments at the Estonian site [11].

**Figure 1:**
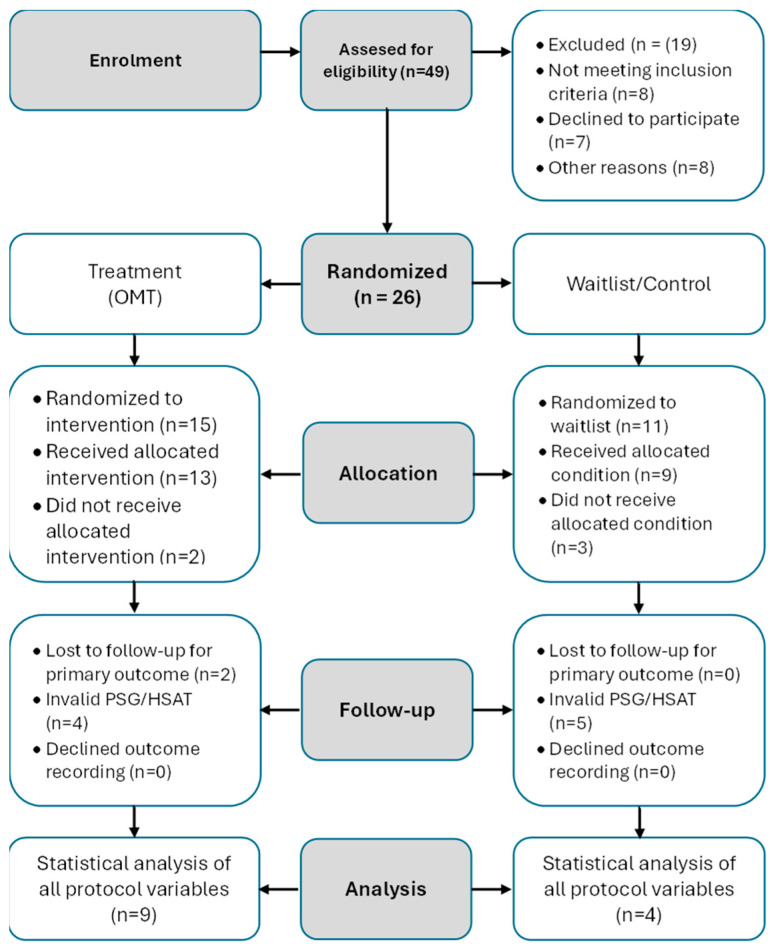
CONSORT flow diagram of participant recruitment, allocation, follow-up, and analysis.

This RCT subsample constituted a pre-specified secondary outcome of the OMTaOSA randomized controlled trial (NCT06079073), which enrolled 104 Norwegian and Estonian adults with mild to moderate OSA across two study sites [11]. The cephalometric analysis was conducted exclusively at the Estonian site (*n* = 49 assessed for eligibility) due to the availability of cone-beam computed tomography (CBCT) equipment and certified radiology personnel. Of these, 26 Estonian participants were randomized (15 to OMT, 11 to waitlist), and 13 participants completed both baseline and follow-up assessments with valid CBCT imaging and polysomnography data (9 OMT and 4 waitlist). During follow-up, 2 participants in the OMT group were lost before the primary outcome assessment. In addition, 4 sleep recordings in the OMT group were excluded because of invalid polysomnography or home sleep apnea testing data. In the waitlist group, no participants were lost to follow-up; however, 5 sleep recordings were excluded due to technical insufficiency. From the total randomized cohort, a dedicated Estonian imaging subsample was formed for craniofacial and hyoid bone analysis. This subsample comprised 13 participants (4 females and 9 males; mean age 38.7 ± 6.2 years), of whom 9 were allocated to the OMT group and 4 to the waitlist control group. All participants in this subsample underwent assessments at baseline (T1) and after 12 weeks of follow-up (T2). Imaging assessments included cone-beam computed tomography (CBCT) for craniofacial and hyoid bone measurements, as well as home-based sleep recordings, as detailed below. Participant recruitment, allocation, follow-up, and analysis are summarized in the CONSORT flow diagram (Figure 1).

### 2.4. Outcome Measurements

This study represents an exploratory CBCT imaging substudy within the larger OMTaOSA randomized controlled trial. The primary outcome of the present substudy was the change in hyoid bone position following orofacial myofunctional therapy. Sleep parameters, including the apnea–hypopnea index (AHI), were analyzed as secondary exploratory outcomes to assess potential associations between structural and functional changes.

#### 2.4.1. Cephalometric and Hyoid Imaging Outcomes (Primary Outcomes)

Pre- and post-intervention CBCT scans were obtained using a NewTom VGi evo X-ray system (Cefla s.c., Imola, Italy) (110 kV, 3 mA, 1.8 s, voxel size 0.3 mm). Cephalograms and superimpositions were generated using Planmeca Romexis^®^ software, version 6.4.5.202 (Planmeca Oy, Helsinki, Finland), and cephalometric analyses were performed using Dolphin Management Premium software (version 11.95; Dolphin Imaging & Management Solutions, Chatsworth, CA, USA). All scans were acquired by a certified radiology technician and independently evaluated by blinded orthodontic residents.

**Figure 2:**
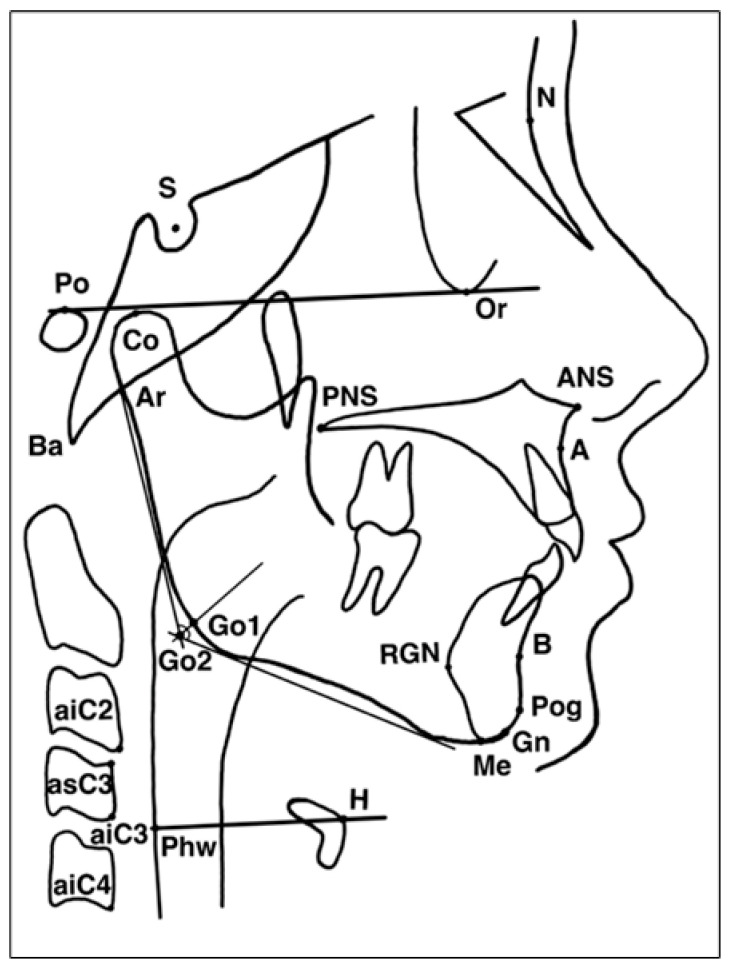
Cephalometric landmarks used for upper-airway and hyoid measurements. Notes: N = nasion; S = sella; Po = porion; Or = orbitale; Co = condylion; Ar = articulare; Ba = basion; ANS = anterior nasal spine; PNS = posterior nasal spine; A = point A (subspinale); B = point B (supramentale); Pog = pogonion; Gn = gnathion; Me = menton; RGN = retrognathion; Go1/Go2 = gonion (superior/inferior); H = hyoidale; Phw = posterior pharyngeal wall; aiC2, aiC3, aiC4 = anterior–inferior points of cervical vertebrae C2 to C4; as C3 = anterior–superior point of C3.

**Figure 3:**
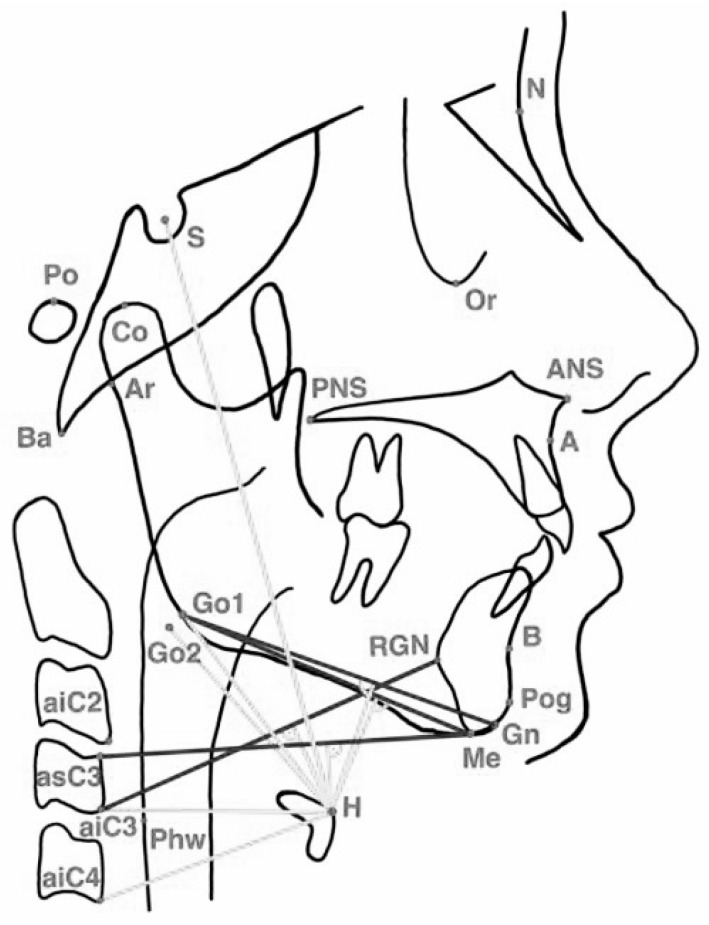
Linear and Angular Cephalometric Measurements. Notes: Distances and angles used for cephalometric analysis. Linear and angular measurements illustrating the spatial relationships among the mandible, hyoid bone, cranial base, posterior pharyngeal wall, and cervical spine. Linear distances measured were H–RGN, H–Go1, H–Go2, H–Phw, RGN–Go1, RGN–Go2, Ba–H, ANS–H, PNS–H, H–aiC2, H–aiC3, and H–aiC4. Angular measurements assessed were ∠Go1–H–RGN, ∠Go2–H–RGN, ∠Ba–H–RGN, and ∠H–aiC3–asC3. Solid lines denote linear measurements, and intersecting line pairs denote angular constructions.

The cephalometric analysis included 26 reference landmarks and 49 cephalometric variables, with a primary focus on hyoid bone position, mandibular relationships, and upper-airway dimensions (Figure 2). The linear and angular measurements used for the cephalometric evaluation are illustrated in (Figure 3). Detailed definitions of the cephalometric landmarks used in the hyoid and craniofacial analysis are provided in Appendix A. An overview of the hyoid musculature and its functional relevance to upper-airway physiology is presented in Appendix B.

#### 2.4.2. Sleep Study Outcomes (Secondary Outcomes)

All participants underwent three consecutive nights of self-applied polysomnography (PSG) using the Nox SAS system (Nox Medical, Reykjavik, Iceland) at baseline and at the 12-week follow-up. Recordings were scored by a certified sleep technologist according to the American Academy of Sleep Medicine (AASM) scoring criteria using Noxturnal Research software (version 6.1.0.30257, Nox Medical, Reykjavik, Iceland).

To account for night-to-night variability, the sleep outcome measures—apnea–hypopnea index (AHI), oxygen desaturation index (ODI), and mean nocturnal SpO_2_—were calculated as the arithmetic mean of the three recorded nights at each time point (T1 and T2). Only recordings meeting predefined technical quality criteria were included in the final statistical analysis.

### 2.5. Intervention

Participants randomized to the intervention group received individualized instruction at baseline and access to a secure digital platform hosting the orofacial myofunctional therapy (OMT) program. Access to the exercise module was granted via a unique code provided in the randomization envelope. The module contained instructional videos for each exercise, a structured daily training schedule, and a calendar for biweekly video consultations with an Academy of Orofacial Myofunctional Therapy to certified therapist. Participants in the control group were granted access to the same digital platform but without the exercise module.

At each study site, a single 60 min in-person introductory session was held to demonstrate the correct execution of the OMT exercises and to provide guidance on using the digital platform. All participants received standardized training materials, including a disposable toothbrush and balloons for use during the exercise period.

Following the introductory session, participants in the intervention group engaged in biweekly video consultations (20–30 min each) over a 12-week intervention period. These sessions were used to review exercise technique, monitor adherence, and guide individualized progression. Adherence monitoring was conducted according to the main trial protocol; however, adherence outcomes are reported separately for the full multicenter cohort and are not a primary focus of the present imaging substudy.

The OMT protocol comprised ten targeted exercises designed to strengthen and coordinate the tongue, soft palate, and facial muscles, thereby enhancing upper-airway patency and neuromuscular control.

#### 2.5.1. Tongue Exercises

The OMT protocol included the following tongue exercises, performed three times daily unless otherwise specified:

- Tongue brushing: Participants brushed the top and sides of the tongue while holding different positions. The movement was repeated five times per session.
- Tongue sliding: Participants placed the tongue tip against the hard palate just posterior to the maxillary incisors and slid it posteriorly along the palate. This movement was repeated 20 times per session.
- Tongue suction: Participants pressed the entire dorsum of the tongue firmly against the hard palate and maintained the contraction for 1 s before relaxing. The protocol also included sustained 10 s holds. Each participant completed 20 short-duration holds and 20 long-duration holds per session
- Tongue depression: Participants maintained tongue tip contact with the mandibular incisors while pressing the posterior tongue downward toward the mouth floor. This exercise was repeated 20 times per session.

#### 2.5.2. Soft Palate Exercises

Participants performed each exercise three times daily.

- Palatal elevation: Participants elevated the soft palate and uvula while phonating “ah,” alternating between short (approximately 1 s) and long “a-a-a” (approximately 5 s) vocalizations. This exercise was performed 20 times per session.
- Balloon inflation: Participants inhaled nasally and exhaled forcefully into a balloon five consecutive times without removing it from the mouth. This sequence was completed three times per session. If a balloon was unavailable, participants performed steady exhalation through pursed lips as an alternative.

#### 2.5.3. Facial Exercises

Participants performed each exercise three times daily.

- Cheek resistance: Participants placed one finger intraorally against the buccal mucosa and contracted the cheek muscles outward against manual resistance. This exercise was performed 10 times per side per session. When intraoral finger placement was not socially feasible during the daytime, the exercise was performed in the morning and evening only
- Air pumping: Participants inflated one cheek with air while maintaining lip seal and then transferred the air from one cheek to the other. This action was repeated 10 times per side per session

This exercise protocol was a modified version of that originally described by Guimarães et al. (2009) [3] and was further refined based on findings from a pilot study conducted at Akershus University Hospital [12]. All exercises were pre-tested for social acceptability and practical feasibility. Participants performed the exercises at home three times daily, for a total of approximately 30–40 min per day over a 12-week period. Instructional videos, produced by the research team, were integrated into the secure digital exercise module.

Progress, adherence, and subjective sleep parameters were assessed during the biweekly video consultations. Training and calibration of the therapists were standardized through joint online or in-person sessions with a senior OMT specialist, both prior to and throughout the study.

### 2.6. Statistical Analysis

Data were collected between September 2023 and January 2025. Due to the small sample size and non-normal data distribution, non-parametric statistical tests were applied. Within-group comparisons (baseline vs. post-intervention) were performed using the Wilcoxon signed-rank test, and between-group differences were analyzed using the Mann–Whitney U test. Baseline-adjusted between-group comparisons were additionally performed using ANCOVA on change scores where appropriate.

Associations between changes in morphometric variables and sleep-related parameters (apnea–hypopnea index, oxygen desaturation index, and mean nocturnal SpO_2_) were evaluated using Kendall’s tau-b correlation as the primary measure due to the small sample size. Pearson’s r correlations were also calculated for comparison. A two-tailed *p*-value < 0.05 was considered statistically significant.

Measurement reliability was assessed by independent, blinded re-evaluation of a random subset of images by both raters. The technical error of measurement (TEM) and percentage TEM (%TEM) were calculated to estimate random error. Intra- and inter-rater reliability were evaluated using intraclass correlation coefficients (intra-rater: ICC (3, 1); inter-rater: ICC (2, 1)). Agreement and potential systematic bias were further assessed using Bland–Altman plots (mean difference ± 1.96 SD) [13].

## 3. Results

At baseline, no significant differences were found between the OMT group (*n* = 9) and the waiting list control group (*n* = 4) across demographic, sleep, or cephalometric variables (all *p* > 0.05). Participants were middle-aged and predominantly male, with similar BMI values in both groups (26.4 vs. 24.4 kg/m^2^; *p* = 0.441).

Sleep parameters were also comparable. Median AHI was 15.1 vs. 11.2 events/h (*p* = 0.767), while ODI (6.8 vs. 8.8 events/h; *p* = 0.314) and mean nocturnal SpO_2_ (94.9% vs. 94.4%; *p* = 0.515) did not differ significantly. The distribution of OSA severity categories was likewise similar (*p* = 0.390). Cephalometric measurements of hyoid position (Hlpaw, MPH, HRGN, C3H, HC3Me) showed no significant between-group differences at baseline (all *p* ≥ 0.123). Baseline demographic, sleep, and cephalometric characteristics are summarized in Table 1.

### 3.1. Within-Group Changes in Sleep and Hyoid Measurements

Within-group analyses showed no statistically significant changes in sleep parameters or core hyoid position measurements in either study group (Table 2). In the therapy group, AHI, ODI, and mean nocturnal SpO_2_ remained stable, with all *p*-values > 0.05. No significant within-group changes were observed in key cephalometric variables (Hlpaw, MPH, HRGN, C3H, HC3Me; all *p* > 0.05). Similarly, the waiting list group demonstrated no statistically significant within-group changes in sleep parameters or hyoid position (all *p* > 0.05).

### 3.2. Between-Group Comparisons of Sleep and Hyoid Position Changes

Between-group analyses revealed no statistically significant differences in primary sleep outcomes between the therapy and waiting list groups, with comparable changes in AHI, ODI, and mean change in nocturnal SpO_2_ (all *p* > 0.05; Table 3).

Several secondary hyoid position measures demonstrated statistically significant between-group differences, with the waiting list group showing markedly greater posterior–inferior hyoid displacement. Large effect sizes were observed (*p* < 0.05, r = 0.56–0.66). Other cephalometric variables showed no statistically significant differences, although several demonstrated trend-level moderate effects favoring greater variability in the waiting list group.

### 3.3. Correlations Between Changes in Hyoid Position and Sleep Parameters

In the overall sample (*n* = 13; Table 4), several hyoid-related variables showed statistically significant associations with sleep-disordered breathing parameters. Changes in mandibular plane-to-hyoid-related measures (ΔHMP2MeGo2Perp) and hyoid-to-nasion distance (ΔHna) were significantly correlated with changes in AHI (τ = 0.503, *p* = 0.017 and τ = 0.452, *p* = 0.032, respectively). In addition, changes in the hyoid-to-C3 distance (ΔHMeasC3withhelper) and hyoid–to–pharyngeal wall distance (ΔHPhw) were significantly correlated with changes in nocturnal oxygen saturation (τ = −0.44, *p* = 0.038 and τ = −0.46, *p* = 0.028, respectively).

In the therapy group (TG, *n* = 9; Table 5), an increase in lower pharyngeal airway width (ΔHlpaw; distance from the hyoid to the posterior pharyngeal wall) was associated with a reduction in AHI (τ = −0.61, *p* = 0.022). Significant correlations with AHI were also observed for changes in mandibular plane–to–hyoid distance (ΔMPH; τ = 0.72, *p* = 0.007), posterior hyoid displacement relative to mandibular landmarks (ΔHMPMeGo1Perp, ΔHMP2MeGo2Perp, ΔHGo1Gn, ΔHGo2Gn), and angular displacement (ΔAngleGnGo2H), with τ values ranging from 0.56 to 0.72 (all *p* ≤ 0.037). Changes in ΔHPhw were significantly correlated with changes in nocturnal oxygen saturation (τ = −0.61, *p* = 0.022).

In the waiting list group (WL, *n* = 4; Table 6), no statistically significant correlations were observed between changes in hyoid position and sleep parameters (all *p* ≥ 0.174). Several correlation coefficients reached high absolute τ values (up to |τ| = 0.67); however, these did not reach statistical significance due to the very small sample size.

### 3.4. Correlations Between Changes in Sleep Parameters and Hyoid Morphometry

Kendall’s tau-b correlations were used to examine the associations between structural craniofacial changes and changes in sleep-disordered breathing indices (AHI, ODI, SpO_2_). In the full sample, several variables demonstrated significant associations with increased AHI, including ΔHMP2MeGo2Perp (τ = 0.50, *p* = 0.017), ΔHGo2Gn (τ = 0.48, *p* = 0.024), and ΔHna (τ = 0.45, *p* = 0.032). Negative correlations were observed between ΔHMeasC3withhelper and SpO_2_ (τ = −0.44, *p* = 0.038), as well as between ΔHPhw and SpO_2_ (τ = −0.46, *p* = 0.028).

When analyses were stratified by treatment group, significant associations were primarily observed in the therapy group. Moderate-to-strong positive correlations with higher AHI values were found for ΔMPH (τ = 0.72, *p* = 0.007), ΔHMPMeGo1Perp (τ = 0.56, *p* = 0.037), ΔHMP2MeGo2Perp (τ = 0.59, *p* = 0.028), ΔHGo1Gn (τ = 0.61, *p* = 0.022), ΔHGo2Gn (τ = 0.67, *p* = 0.012), and ΔHna (τ = 0.72, *p* = 0.007). In addition, increases in hyoid–mandibular distance (ΔHPhw) were negatively associated with SpO_2_ (τ = −0.61, *p* = 0.022). No consistent or statistically significant associations were observed in the waiting list group, indicating the absence of intervention-related correlations (Table 7).

## 4. Discussion

This randomized controlled trial examined the short-term effects of orofacial myofunctional therapy (OMT) on hyoid bone position in adults with mild to moderate obstructive sleep apnea. Although AHI represents the primary clinical endpoint of the parent OMTaOSA trial, this imaging substudy was not powered to detect treatment-related effects on sleep parameters. Accordingly, observed associations between hyoid position changes and sleep outcomes should be interpreted as exploratory.

Observed associations between hyoid position changes and sleep outcomes should therefore be interpreted as exploratory. A key methodological strength of the study was the use of an axis-based analytical framework. In this approach, the hyoid position was quantified using vector-based cephalometric analysis of three-dimensional CBCT imaging. This method enabled the distinction between clinically favorable and unfavorable directional changes in hyoid position. It also provided a more functionally meaningful interpretation of treatment-related structural adaptations than conventional linear measurements and offered greater insight into the biomechanical implications of OMT.

**Figure 4:**
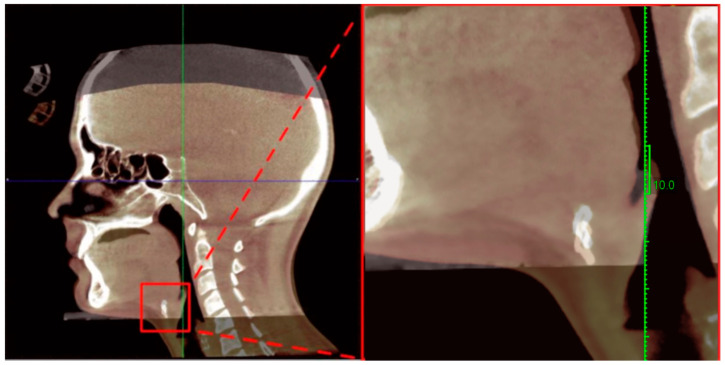
CBCT-based lateral cephalometric superimposition of a representative participant illustrating hyoid bone position before and after the 12-week intervention. Notes. Lateral cephalometric CBCT superimpositions of representative participants illustrating hyoid bone position before and after the 12-week intervention. The baseline position (T1) is shown in gray and the post-intervention position (T2) in white. The hyoid region is marked by the box and magnified in the adjacent panel. Superimposition was performed on stable cranial base and cervical vertebral reference structures. The images demonstrate small-magnitude but directionally consistent changes in hyoid position, primarily reflecting cranial stabilization following orofacial myofunctional therapy.

The principal finding was that hyoid position remained stable or showed modest cranial displacement in the OMT group. In contrast, the waiting list group demonstrated greater posterior–inferior displacement. The principal finding was that hyoid position remained stable or showed modest cranial displacement in the OMT group (Figure 4). Despite this structural stabilization, no statistically significant changes were observed in polysomnographic parameters, including the apneatohypopnea index (AHI), oxygen desaturation index (ODI), or mean nocturnal SpO_2_, and the associations between changes in hyoid position and sleep indices were weak. These findings are consistent with previous evidence indicating that the clinical efficacy of OMT is more pronounced with long-term, high-frequency intervention protocols, whereas shorter interventions often result in subclinical effects [3,5,7]. Notably, a recent study demonstrated that even approximately 10 min daily exercise sessions can reduce AHI when patient adherence is sustained, suggesting that treatment duration and consistency may be more critical than the intensity of individual sessions [14].

The stabilization of hyoid position in the OMT group may reflect preserved neuromuscular tone in the suprahyoid and genioglossus muscles. In contrast, the greater positional variability in the waiting list group may represent natural positional changes or progressive decompensation of upper-airway musculature. These observations are consistent with findings from cephalometric studies. Those studies show that inferior or posterior hyoid positioning is associated with greater OSA severity and lower minimum oxygen saturation [10,15,16,17,18]. According to the hyoid slump hypothesis, caudal descent of the hyoid represents a key mechanism through which aging, increased body weight, and reduced muscle tone contribute to heightened susceptibility to upper airway collapse [9].

From a therapeutic perspective, it is essential to define which hyoid displacement vectors can be considered favorable. In this study, favorable effects were defined as positional changes associated with reduced disease burden, particularly widening of the lower pharyngeal airway (chg_Hlpaw) and prevention of an unfavorable increase in the hyoid–gonion distance (chg_HGo2Gn). Although between-group differences in ΔHlpaw did not reach statistical significance, its negative association with AHI suggests that early structural effects of OMT may be more evident in oxygenation-related indices than in AHI alone.

Increasing evidence indicates that ODI and oxygen desaturation metrics capture OSA severity and cardiovascular risk more sensitively than AHI, particularly in short-term interventions [19,20]. Accordingly, ODI and SpO_2_ may represent earlier and more responsive markers of treatment-related change than AHI in short-duration OMT protocols. The absence of concomitant improvement in AHI or SpO_2_ despite hyoid stabilization likely reflects the relatively short intervention duration, the small magnitude of structural change, limited statistical power, and the use of static measurements that do not capture dynamic airway behavior during sleep. Similar dissociations between early structural adaptation and delayed functional improvement have been reported in previous OMT studies [3,7,20,21]. Favorable response patterns were associated with activation of the anterior tongue, increased tongue–palate contact, and engagement of the suprahyoid musculature, promoting a cranial and/or anterior orientation of the hyoid. These mechanisms are supported by mechanistic and clinical studies demonstrating that tongue and soft palate exercises reduce retropalatal collapse and enhance upper airway dilator muscle function [5,22,23]. Biomechanical and imaging studies indicate that both hyoid position and displacement vectors critically influence airway patency. Three-dimensional MRI and finite element modeling demonstrate that cranial and anterior hyoid displacement improves airway openness, whereas caudal displacement increases collapsibility even when other morphometric parameters appear favorable [21,24,25]. Thus, the hyoid stabilization observed in the OMT group is biomechanically meaningful despite the absence of short-term AHI improvement and may be particularly relevant in mild OSA or as part of multimodal treatment strategies combining OMT with mandibular advancement or orthodontic interventions [10,15,16,17,18].

The axis-based analysis indicated that, in some patients, certain exercises may promote posterior tongue displacement or approximation of the hyoid toward the C3 level. Such displacement patterns have been associated with lower minimum SpO_2_ and more severe sleep apnea [18,26]. Therefore, examining correlations alone is insufficient; it is essential to determine whether treatment prevents an unfavorable directional shift or instead amplifies it. This supports the use of axis-based analysis as a valuable complement to conventional cephalometry.

Not all OMT-induced changes are necessarily therapeutically beneficial, and interpretation based solely on increases or decreases in individual indices—particularly in 2D analyses—may be misleading. A single linear measurement cannot reliably represent the true spatial displacement of the hyoid, which requires simultaneous assessment of vertical and horizontal components. For example, an increase in the H–C3 distance (H3C) may indicate either cranial displacement toward the mandible or caudal movement toward the cervical spine, depending on its relationship to other reference structures. Therefore, changes in isolated indices must be interpreted in conjunction with mandibular and vertical reference measures (e.g., MPH) to determine the true direction of hyoid movement. In the present study, axis-based analysis, using a coordinate system to define spatial position, enabled differentiation between cranial and caudal as well as anterior and posterior displacement vectors. This approach revealed that in some patients, both H3C and MPH increased simultaneously, indicating true caudal displacement, which is biomechanically unfavorable. Such displacement patterns have been associated with lower minimum SpO_2_ and greater OSA severity [18,26]. Accordingly, correlation analysis alone is insufficient; evaluation of directional vectors is essential to determine whether treatment prevents or amplifies unfavorable displacement. These findings support the use of axis-based analysis as a critical complement to conventional cephalometry.

Longitudinal CBCT imaging was performed at a single study site only, as only this cohort underwent both pre- and post-intervention imaging. This restriction prevented full utilization of the multicenter design for structural outcomes and limited the sample size of the imaging substudy. However, this RCT imaging substudy has several important limitations. The small sample size (*n* = 13) and the unbalanced group allocation (*n* = 9 vs. *n* = 4) limit statistical power and generalizability. In the main trial, the priori sample size calculation for the functional endpoint (AHI) assumed a large effect (Cohen’s d = 0.8), yielding a target of approximately 50 participants per group [11]; thus, the available sample in our setting (*n* = 26 per group) corresponds to approximately 50% of this target. This is in line with prior specialized imaging work in the OMT/MFT field, where cohorts are typically small (e.g., Villa et al., 2015, *n* = 14) [27]. These limitations reflect the current state of the OMT research field, in which randomized controlled trials remain scarce and frequently rely on small samples [23]. Accordingly, this exploratory imaging substudy provides the first quantitative estimates of OMT-induced hyoid position changes and should be interpreted as hypothesis-generating. The main OMTaOSA trial (N = 104, currently in preparation) will provide definitive evidence on functional efficacy with adequate power for AHI analysis [11]. The use of a waiting-list control rather than a sham exercise protocol was chosen due to the difficulty of defining an inert sham in orofacial myofunctional therapy and for ethical considerations in patients with obstructive sleep apnea [3,7,23,27]. However, this design does not control for non-specific effects such as therapist contact or patient expectations. Future studies should explore alternative active control conditions that minimize upper-airway muscle engagement. Exercise performance could be objectively monitored using a digital sleep diary and web-based therapy sessions; however, completion of the diary was time-consuming and burdensome for participants, which may have affected true adherence despite monitoring efforts [3,14]. Although previous studies have shown that structured telemedicine- and app-based OMT programs can be feasible and associated with improvements in subjective sleepiness and patient-reported outcomes, indicating that remote delivery may support treatment engagement [28,29,30], other investigations have highlighted substantial barriers to long-term adherence, including time demands, motivational challenges, and the burden of frequent exercise logging [12,30]. These observations are consistent with the participant feedback in the present study.

In addition, static cephalometry does not capture the dynamic behavior of the hyoid bone and upper airway during sleep. Dynamic techniques such as drug-induced sleep endoscopy (DISE) have demonstrated that obstruction-related movements directly reflect airway mechanics and collapse patterns [25,27,29]. A recent prospective study combining DISE with hyoid ultrasound further showed that the magnitude of hyoid motion during obstructive respiratory events is directly associated with increased respiratory effort, higher baseline hyoid position, and greater pharyngeal collapsibility [25,27]. The authors concluded that caudal hyoid displacement may represent a compensatory response to upper airway obstruction. These findings indicate that static awake measurements may not adequately reflect true hyoid motion vectors during sleep-related collapse. Therefore, future studies should integrate axis-based cephalometric analysis with DISE, dynamic MRI, or real-time ultrasound for a more comprehensive functional assessment.

An additional limitation relates to the web-based nature of the intervention. Although initial instruction was provided during an in-person visit, subsequent therapy was delivered primarily through online sessions. Participant feedback indicated that this approach did not always provide sufficient motivation for consistent or correct exercise performance. Furthermore, the exercise program was perceived by several participants as too long and time-consuming, which may have negatively affected adherence [12,27]. All participants in the intervention group followed the same standardized exercise protocol. It is reasonable to assume that a more personalized OMT program, tailored to individual oropharyngeal muscle tone and specific mechanisms of airway obstruction, could yield superior clinical and structural outcomes compared with a uniform protocol [12].

Although adherence to the OMT protocol was prospectively monitored using exercise diaries and scheduled online consultations as part of the main trial protocol, detailed quantitative adherence data were not available for robust subgroup analysis within this pilot imaging substudy. Attrition during the later stages of follow-up and incomplete datasets limited adherence reporting in the Estonian subsample. Given the known impact of adherence on OMT outcomes, this represents an additional limitation and should be considered when interpreting the results. Future studies should therefore implement longer-duration, standardized OMT protocols and incorporate objective adherence monitoring. In addition, OMT should be more clearly positioned within a phenotype-based multimodal treatment framework. In this model, myofunctional therapy is combined with CPAP therapy, oral appliances, orthodontic interventions, and, when indicated, surgical procedures [7,18,25].

## 5. Conclusions

Short-term orofacial myofunctional therapy did not result in significant improvements in objective sleep-related outcomes. However, it was associated with altered hyoid biomechanics compared with the waiting list group. These findings suggest a potential early structural effect of therapy that may precede measurable functional improvement.

In addition, the direction-based analysis of hyoid positional changes introduced in this study appears to provide a sensitive methodological framework for assessing subtle biomechanical treatment effects using cephalometric measurements. This approach may contribute to future standardization of hyoid position assessment in interventional studies. Larger studies with longer follow-up and objective adherence monitoring are warranted to clarify the clinical relevance of these changes and to further validate the proposed methodology.

## Figures and Tables

**Table 1:** Baseline Characteristics and Sleep Parameters.

Characteristic	Therapy (*n* = 9)	Waiting List (*n* = 4)	*p*-Value
Age, years	45.0 (38.0–52.0)	48.5 (42.0–55.0)	0.45
Male, *n* (%)	7 (77.8%)	3 (75.0%)	0.999
BMI, kg/m^2^	26.4 (21.9–28.9)	24.4 (22.2–26.4)	0.441
Neck circumference, cm	37.5 (35.5–42.5)	35.5 (32.0–40.9)	0.276
Sleep parameters			
AHI, events/h	15.1 (10.1–17.9)	11.2 (5.0–17.8)	0.767
ODI, events/h	6.8 (4.6–10.3)	8.8 (2.5–14.9)	0.314
SpO_2_, %	94.9 (94.0–95.3)	94.4 (93.3–94.8)	0.515
AHI severity, *n* (%)			
Mild (5–14.9)	1 (11.1%)	1 (25.0%)	0.39
Moderate (15–29.9)	3 (33.3%)	2 (50.0%)	
Severe (≥30)	5 (55.6%)	1 (25.0%)	
Core hyoid measurements			
Hlpaw, mm	9.6 (8.1–12.3)	8.5 (4.9–14.4)	0.123
MPH, mm	19.8 (18.7–21.9)	12.8 (11.7–24.1)	0.515
HRGN, mm	37.5 (32.0–39.3)	37.5 (29.1–44.3)	0.515
C3H, mm	36.9 (34.2–39.8)	34.7 (28.2–42.3)	0.575
HC3Me, mm	9.4 (6.6–12.7)	2.5 (3.9–7.2)	0.953

Notes: Data presented as median (interquartile range) or *n* (%). BMI = body mass index; AHI = Apnea–Hypopnea Index; ODI = Oxygen Desaturation Index; SpO_2_ = peripheral oxygen saturation; Hlpaw = hyoid to lower posterior airway wall; MPH = mandibular plane to hyoid; HRGN = hyoid to retrognathion; C3H = C3 vertebra to hyoid; HC3Me = hyoid-C3-menton distance. *p*-values from Mann–Whitney U test or Fisher’s exact test.

**Table 2:** Within-Group Changes in Sleep Parameters and Core Hyoid Measurements.

Variable	Baseline (TG)	Post (TG)	Change (TG)	*p* (TG)	Baseline (WL)	Post (WL)	Change (WL)	*p* (WL)
AHI, events/h	15.1 (10.1–17.9)	13.9 (8.0–17.1)	−1.2 (−3.5–0.8)	0.296	11.2 (5.0–17.8)	13.8 (3.8–25.2)	+2.9 (−0.5–7.5)	0.422
ODI, events/h	6.8 (4.6–10.3)	8.0 (4.1–11.8)	+0.9 (−0.6–2.2)	0.314	8.8 (2.5–14.9)	7.7 (1.5–18.0)	+0.3 (−1.5–1.8)	0.463
SpO_2_, %	94.9 (94.0–95.3)	94.8 (93.8–95.1)	−0.05 (−0.30–0.20)	0.515	94.4 (93.3–94.8)	93.7 (93.4–94.9)	−0.14 (−0.50–0.30)	0.600
Hlpaw, mm	9.6 (8.1–12.3)	10.4 (7.3–14.3)	+0.8 (−0.3–2.0)	0.123	8.5 (4.9–14.4)	10.0 (7.5–12.2)	+0.6 (−1.2–4.6)	0.208
MPH, mm	19.8 (18.7–21.9)	19.9 (17.5–22.8)	+0.3 (−0.7–1.5)	0.515	12.8 (11.7–24.1)	13.1 (11.5–22.8)	−0.4 (−1.0–0.5)	0.701
HRGN, mm	37.5 (32.0–39.3)	37.5 (33.2–39.0)	−0.2 (−1.5–1.3)	0.515	37.5 (29.1–44.3)	35.4 (25.0–38.1)	−4.2 (−7.2–1.1)	0.075
C3H, mm	36.9 (34.2–39.8)	37.0 (34.3–38.8)	+0.4 (−0.8–1.6)	0.575	34.7 (28.2–42.3)	35.3 (29.3–41.2)	+0.2 (−1.4–1.8)	0.583
HC3Me, mm	9.4 (6.6–12.7)	8.4 (6.2–12.4)	−0.1 (−1.2–1.0)	0.953	2.5 (−3.9–7.2)	6.7 (−4.0–10.7)	+2.6 (−0.5–5.4)	0.382

Notes: Data are presented as median (interquartile range). Changes are expressed as median change (interquartile range). Within-group comparisons were performed using the Wilcoxon signed-rank test. TG, therapy group; WL, waiting list; AHI, apnea–hypopnea index; ODI, oxygen desaturation index; SpO_2_, mean nocturnal oxygen saturation; Hlpaw, horizontal distance from hyoid to posterior airway wall; MPH, mandibular plane to hyoid distance; HRGN, hyoid to retrognathion distance; C3H, third cervical vertebra to hyoid distance; HC3Me, hyoid to C3–menton distance.

**Table 3:** Between-Group Comparisons of Changes in Sleep and Hyoid Parameters.

Variable	Therapy Group Change (*n* = 9)	Waiting List Change (*n* = 4)	U	*p*-Value	r	Clinical Interpretation
Sleep parameters						
ΔAHI, events/h	−1.2 (−3.5–0.8)	+2.9 (−0.5–7.5)	12.0	0.767	−0.08	No significant difference
ΔODI, events/h	+0.9 (−0.6–2.2)	+0.3 (−1.5–1.8)	15.0	0.314	−0.28	No significant difference
ΔSpO_2_, %	−0.05 (−0.30–0.20)	−0.14 (−0.50–0.30)	15.0	0.515	−0.18	No significant difference
Hyoid position						
ΔHlpaw, mm	+0.8 (−0.3–2.0)	+0.6 (−1.2–4.6)	13.0	0.123	−0.43	No significant difference
ΔMPH, mm	+0.3 (−0.7–1.5)	−0.4 (−1.0–0.5)	14.0	0.515	−0.18	No significant difference
ΔHRGN, mm	−0.2 (−1.5–1.3)	−4.2 (−7.2–1.1)	4.0	0.031	−0.60	Large effect; WL decreased more
ΔC3H, mm	+0.4 (−0.8–1.6)	+0.2 (−1.4–1.8)	16.0	0.575	−0.16	No significant difference
ΔHC3Me, mm	−0.1 (−1.2–1.0)	+2.6 (−0.5–5.4)	11.0	0.090	−0.47	Moderate effect; trend
Significant additional changes						
ΔAngleGnGo2H, °	−0.2 (−2.0–1.8)	−4.8 (−13.9–3.4)	8.0	0.090	−0.47	Moderate effect; trend
ΔHRgn1_A, mm	−0.3 (−1.2–0.6)	−2.3 (−4.8–0.0)	5.0	0.031	−0.60	Large effect; WL decreased more
ΔHGo1, mm	+0.5 (−1.0–2.2)	+7.9 (2.0–13.0)	3.0	0.045	−0.56	Large effect; WL increased more
ΔHGo2, mm	−1.6 (−3.5–0.8)	+7.9 (3.1–12.7)	1.0	0.017	−0.66	Large effect; WL increased more
ΔHMe, mm	+0.2 (−2.5–2.9)	−4.2 (−8.8–0.4)	2.5	0.063	−0.52	Moderate effect; trend
ΔAngleBMeHy, °	+0.9 (−1.5–3.2)	+4.1 (1.5–6.8)	2.5	0.064	−0.51	Moderate effect; trend

Notes: Data are presented as median (interquartile range). Δ denotes change from baseline to follow-up. Between-group comparisons were performed using the Mann–Whitney U test. U, Mann–Whitney U statistic; r, effect size (Z/√N). Bold *p*-values indicate statistical significance at *p* < 0.05. Negative r values indicate a greater magnitude of change in the waiting list group. Abbreviations: AHI, apnea–hypopnea index; ODI, oxygen desaturation index; SpO_2_, peripheral oxygen saturation; Hlpaw, distance from hyoid to lower posterior airway wall; MPH, mandibular plane–to–hyoid distance; HRGN, hyoid–to–retrognathion distance; C3H, C3 vertebra–to–hyoid distance; HC3Me, hyoid–C3–menton distance; AngleGnGo2H, gonion–gnathion–hyoid angle; HRgn1_A, horizontal retrognathion–to–hyoid distance; HGo1, hyoid–to–gonion linear distance; HGo2, second gonion–to–hyoid linear distance; HMe, hyoid–to–menton distance; AngleBMeHy, angle between B-point, menton, and hyoid.

**Table 4:** Correlations (Kendall’s τ) between Changes in Hyoid Position and Sleep Parameters (Overall, *n* = 13).

Variable	ΔAHI τ	*p*	ΔODI τ	*p*	ΔSpO_2_ τ	*p*
ΔHlpaw	−0.36	0.088	−0.10	0.625	0.21	0.329
ΔMPH	0.41	0.051	0.26	0.222	−0.15	0.464
ΔHMPMeGo1Perp	0.41	0.057	0.22	0.297	−0.22	0.297
ΔHMP2MeGo2Perp	0.50	0.017	0.25	0.246	−0.30	0.160
ΔHGo1Gn	0.41	0.051	0.26	0.222	−0.26	0.222
ΔHGo2Gn	0.48	0.024	0.17	0.427	−0.40	0.058
ΔHMeasC3withhelper	0.28	0.180	0.23	0.272	−0.44	0.038
ΔHna	0.45	0.032	−0.12	0.582	0.07	0.760
ΔHApoint	0.28	0.180	0.03	0.903	−0.08	0.714
ΔHRgn1_A	0.22	0.299	0.12	0.582	−0.07	0.760
ΔHPhw	0.00	0.999	0.21	0.329	−0.46	0.028
ΔAngleGnGo2H	0.36	0.088	0.26	0.222	−0.31	0.143

Notes: Data are presented as Kendall’s τ correlation coefficients with two-tailed *p*-values. Δ indicates change from baseline to follow-up. Correlation analyses were performed using Kendall’s tau-b test. *p* < 0.05 was considered statistically significant. Abbreviations: AHI, apnea–hypopnea index; ODI, oxygen desaturation index; SpO_2_, peripheral oxygen saturation; ΔHlpaw, change in the distance from the hyoid to the posterior pharyngeal wall; ΔMPH, change in the mandibular plane–to–hyoid distance; ΔHMPMeGo1Perp, change in the perpendicular distance from the hyoid to the mandibular plane (Me–Go1); ΔHMP2MeGo2Perp, change in the second perpendicular mandibular plane–to–hyoid distance; ΔHGo1Gn, change in the hyoid–to–gonion–gnathion distance; ΔHGo2Gn, change in the second hyoid–to–gonion–gnathion distance; ΔHMeasC3withhelper, change in the hyoid–to–C3 distance measured with helper reference; ΔHna, change in the hyoid–to–nasion distance; ΔHApoint, change in the hyoid–to–A point distance; ΔHRgn1_A, change in the horizontal retrognathion–to–hyoid distance; ΔHPhw, change in the hyoid–to–pharyngeal airway width; ΔAngleGnGo2H, change in the gonion–gnathion–hyoid angle.

**Table 5:** Correlations (Kendall’s τ) between Changes in Hyoid Position and Sleep Parameters—Therapy Group (*n* = 9).

Variable	ΔAHI τ	*p*	ΔODI τ	*p*	ΔSpO_2_ τ	*p*
ΔHlpaw	−0.61	0.022	−0.06	0.835	0.39	0.144
ΔMPH	0.72	0.007	0.06	0.835	−0.06	0.835
ΔHMPMeGo1Perp	0.56	0.037	0.11	0.677	−0.33	0.211
ΔHMP2MeGo2Perp	0.59	0.028	0.03	0.917	−0.37	0.173
ΔHGo1Gn	0.61	0.022	0.06	0.835	−0.28	0.297
ΔHGo2Gn	0.67	0.012	0.00	0.999	−0.44	0.095
ΔHMeasC3withhelper	0.61	0.022	0.06	0.835	−0.39	0.144
ΔHna	0.72	0.007	−0.06	0.835	−0.17	0.532
ΔHApoint	0.56	0.037	0.00	0.999	−0.33	0.211
ΔHRgn1_A	0.61	0.022	0.17	0.532	−0.39	0.144
ΔHPhw	0.06	0.835	0.06	0.835	−0.61	0.022
ΔAngleGnGo2H	0.72	0.007	0.06	0.835	−0.39	0.144

Notes: Same statistical definitions, tests, and abbreviations as in Table 4.

**Table 6:** Correlations (Kendall’s τ) between Changes in Hyoid Position and Sleep Parameters—Waiting List Group (*n* = 4).

Variable	ΔAHI τ	*p*	ΔODI τ	*p*	ΔSpO_2_ τ	*p*
ΔHlpaw	0.00	0.999	0.00	0.999	0.00	0.999
ΔMPH	0.00	0.999	0.67	0.174	−0.67	0.174
ΔHMPMeGo1Perp	0.67	0.174	0.67	0.174	−0.67	0.174
ΔHMP2MeGo2Perp	0.33	0.497	0.33	0.497	−0.33	0.497
ΔHGo1Gn	0.33	0.497	—	—	—	—
ΔHGo2Gn	0.00	0.999	0.67	0.174	−0.67	0.174
ΔHMeasC3withhelper	0.00	0.999	0.67	0.174	−0.67	0.174
ΔHna	0.00	0.999	−0.67	0.174	0.67	0.174
ΔHApoint	−0.33	0.497	−0.33	0.497	0.33	0.497
ΔHRgn1_A	−0.18	0.718	−0.18	0.718	0.18	0.718
ΔHPhw	0.00	0.999	0.67	0.174	−0.67	0.174
ΔAngleGnGo2H	0.00	0.999	0.67	0.174	−0.67	0.174

Notes: Same statistical definitions, tests, and abbreviations as in Table 4.

**Table 7:** Effects of OMT on Hyoid Position Changes and Sleep Parameters.

Hyoid Parameter	Between-Group Changes			Within-Group Changes		OMT Effect Direction	Sleep Parameter Correlation in Therapy Group		Interpretation
	Waiting Δ	Therapy Δ	*p*-Value	Waiting List	Therapy Group		ΔAHI (τ)	ΔODI (τ)	
ΔC3H	+0.18	+0.36	0.090 †	NS	NS	↑ Increase	−0.083	0.717	Unfavorable: ↑ C3H → ↑ ODI
ΔHlpaw	+0.64	+0.80	NS	NS	NS	↑ Increase	−0.783	−0.133	Favorable: ↑ Hlpaw → ↓ AHI
ΔMPH	−0.37	+0.25	NS	NS	NS	↑ Reversal	0.883	0.067	Unfavorable: ↑ MPH → ↑ AHI
ΔHMPMeGo1Perp	−0.12	+0.32	NS	NS	NS	↑ Reversal	0.733	0.150	Unfavorable: ↑ Distance → ↑ AHI
ΔHMP2MeGo2Perp	+0.45	+0.13	NS	NS	NS	↑ Increase	0.787	−0.025	Unfavorable: ↑ Distance → ↑ AHI
ΔHGo1Gn	+0.15	+0.39	0.045 *	*p* = 0.068 †	NS	↑ Increase	0.750	0.133	Unfavorable: ↑ HGo1Gn → ↑ AHI
ΔHGo2Gn	+0.88	+0.07	0.017 *	*p* = 0.068 †	NS	↓ Prevention	0.783	0.017	Favorable: OMT prevents ↑ HGo2Gn
ΔHMeasC3withhelper	−0.05	+0.44	NS	NS	NS	↑ Increase	0.817	−0.017	Unfavorable: ↑ Distance → ↑ AHI
ΔHna	+0.23	+0.10	NS	NS	NS	↑ Increase	0.883	−0.050	Unfavorable: ↑ Hna → ↑ AHI
ΔHApoint	−1.42	+0.72	NS	NS	NS	↑ Reversal	0.783	0.017	Unfavorable: ↑ HApoint → ↑ AHI
ΔHBpoint	−3.12	+0.33	0.064 †	*p* = 0.068 †	NS	↑ Reversal	0.633 †	0.000	Trend unfavorable
ΔHRgn1_A	−3.95	+0.29	0.031 *	*p* = 0.068 †	NS	↑ Reversal	0.767	0.217	Unfavorable: ↑ HRgn1_A → ↑ AHI

Notes. Data are presented as changes (Δ). Between- and within-group comparisons were analyzed using non-parametric tests. Correlation coefficients are reported as Kendall’s τ due to the small sample size. Pearson’s r correlations were additionally calculated for comparison and showed the same direction and significance pattern. * *p* < 0.05 denotes statistical significance; † trend-level. NS = not significant. OMT effect direction: ↑ increase; ↓ decrease; Reversal = change opposite to the waiting-list group; Prevention = prevention of an adverse increase. Favorable effects indicate hyoid positional changes associated with lower AHI or ODI; unfavorable effects indicate changes associated with higher AHI or OD. The horizontal arrow (→) indicates the association between hyoid position changes and sleep parameter outcomes.

## Data Availability

De-identified numerical datasets are available from the corresponding author upon reasonable request. Due to privacy regulations, CBCT imaging data cannot be publicly shared.

## Appendix A. Definitions of Cephalometric Landmarks Used in Hyoid and Craniofacial Analysis

**Acronym**	**Landmark/Full Term**	**Definition**
N	Nasion	The most anterior point of the fron–nasal suture.
S	Sella	The midpoint of the sella turcica.
Po	Porion	The uppermost point on the external auditory meatus.
Or	Orbitale	The lowest point on the inferior margin of the orbit.
Co	Condylion	The most superior point on the mandibular condyle.
Ar	Articulare	The intersection of the posterior border of the mandibular ramus and the cranial base.
Ba	Basion	The most inferior point on the anterior margin of the foramen magnum.
ANS	Anterior Nasal Spine	The tip of the anterior nasal spine.
PNS	Posterior Nasal Spine	The most posterior point on the hard palate.
A	Point A (Subspinale)	The deepest midline point between the anterior nasal spine and the maxillary alveolar crest.
B	Point B (Supramentale)	The deepest midline point between the alveolar crest and the chin on the mandibular contour.
Pog	Pogonion	The most anterior point on the bony chin.
Gn	Gnathion	The most anterior–inferior point on the mandibular symphysis.
Me	Menton	The lowest point on the mandibular symphysis.
RGN	Retrognathion	The most posterior–inferior point on the bony chin.
Go1/Go2	Gonion (Upper/Lower)	Reference points at the posterior–inferior angle of the mandible used for angular measurements.
H	Hyoidale	The most superior and anterior point on the body of the hyoid bone.
Phw	Posterior Pharyngeal Wall	The posterior wall of the pharynx at the oropharyngeal level.
aiC2, aiC3, aiC4	Anterior–Inferior Points of Cervical Vertebrae C2–C4	The most inferior–anterior points of the respective cervical vertebral bodies.
asC3	Anterior–Superior Point of C3	The most superior–anterior point on the C3 vertebral body.

## Appendix B. Hyoid Musculature: Anatomy and Functional Roles Relevant—Upper Airway Physiology

**Muscle Group/Muscle**	**Primary Actions**	**Relevance—Swallowing, Speech, and Upper Airway Physiology**
Suprahyoid muscles		
Digastric (anterior and posterior bellies)	Elevates hyoid with fixed mandible; depresses mandible with fixed hyoid; contributes—mouth opening and lateral grinding	Assists oral floor elevation during swallowing; influences mandibular and –ngue positioning affecting oropharyngeal airway space
Stylohyoid	Elevates and retracts hyoid; elongates oral floor	Supports tongue elevation and coordinated bolus transit; contributes—upper airway stabilization
Mylohyoid	Elevates hyoid and oral floor with fixed mandible; depresses mandible with fixed hyoid; contributes—lateral grinding	Facilitates oral-phase swallowing and phonation; elevates hyoid and tongue base to help maintain airway patency
Geniohyoid	Elevates and anteriorly displaces hyoid; widens airway; assists grinding	Enhances airway patency through anterior hyoid movement; important in swallow initiation and upper airway stabilization
Infrahyoid muscles		
Omohyoid (superior and inferior bellies)	Depresses and stabilizes hyoid; tenses cervical fascia; maintains internal jugular vein patency	Stabilizes hyoid during respiration; coordinates hyoid–laryngeal motion impacting airway diameter
Sternohyoid	Depresses and stabilizes hyoid during mandibular movements	Helps maintain lower hyoid position, influencing tongue base posture and airway configuration
Sternothyroid	Depresses and stabilizes larynx during phonation	Modulates laryngeal height, airflow characteristics, and airway resistance
Thyrohyoid	Elevates larynx with fixed hyoid; depresses hyoid with fixed larynx	Coordinates laryngeal elevation during swallowing; influences upper airway aperture during phonation and respiration
